# A randomised controlled trial on roles of prostaglandin E1 nebulization among patients undergoing one lung ventilation

**DOI:** 10.1186/s12890-022-01831-4

**Published:** 2022-01-13

**Authors:** Pengyi Li, Lianbing Gu, Jing Tan, Zhenghuan Song, Qingming Bian, Dian Jiao, Zeping Xu, Lijun Wang

**Affiliations:** Department of Anesthesiology, Jiangsu Cancer Hospital and Jiangsu Institute of Cancer Research, The Affiliated Cancer Hospital of Nanjing Medical University, No. 42 Baiziting, Xuanwu District, Nanjing, 210009 Jiangsu China

**Keywords:** Prostaglandin E1, One-lung ventilation, 40% FiO_2_, Randomised controlled trial

## Abstract

**Background:**

Prostaglandin E1 (PGE1) has been reported to maintain adequate oxygenation among patients under 60% FiO_2_ one-lung ventilation (OLV). This research aimed to explore whether PGE1 is safe in pulmonary shunt and oxygenation under 40% FiO_2_ OLV and provide a reference concentration of PGE1.

**Methods:**

Totally 90 esophageal cancer patients treated with thoracotomy were enrolled in this study, randomly divided into three groups (n = 30/group): Group A (60% FiO_2_ and 0.1 µg/kg PGE1), Group B (40% FiO_2_ and 0.1 µg/kg PGE1), and Group C (40% FiO_2_, 0.2 µg/kg PGE1). Primary outcomes were oxygenation and pulmonary shunt during OLV. Secondary outcomes included oxidative stress after OLV.

**Results:**

During OLV, patients in Group C and B had lower levels of PaO_2_, SaO_2_, SpO_2_, MAP, and Qs/Qt than those in Group A (*P* < 0.05). At T2 (OLV 10 min), patients in Group C and B exhibited a lower level of PaO_2_/FiO_2_ than those in Group A, without any statistical difference at other time points. The IL-6 levels of patients in different groups were different at T8 (F = 3.431, *P* = 0.038), with IL-6 in Group C being lower than that in Group B and A. MDA levels among the three groups differed at T5 (F = 4.692, *P* = 0.012) and T7 (F = 5.906, *P* = 0.004), with the MDA level of Group C being lower than that of Group B and A at T5, and the MDA level of Group C and B being lower than that of Group A at T7. In terms of TNF-α level, patients in Group C had a lower level than those in Group B and A at T8 (F = 3.598, *P* = 0.033). Compared with patients who did not use PGE1, patients in Group C had comparable complications and lung infection scores.

**Conclusion:**

The concentration of FiO_2_ could be reduced from 60 to 40% to maintain oxygenation. 40% FiO_2_ + 0.2 µg/kg PGE1 is recommended as a better combination on account of its effects on the inflammatory factors.

**Trial registration:** Chictr.org.cn identifier: ChiCTR1800018288, 09/09/2018.

## Background

Open radical esophagectomy with extended lymph node dissection is vital for treating esophageal cancer (EC), whether EC is early and locally advanced [1, 2]. One-lung ventilation (OLV) has been extensively used throughout thoracic surgery, being conducive to surgical exposure [3]. However, OLV can decrease the partial pressure of arterial oxygen (PaO_2_) and lead to hypoxemia by increasing intrapulmonary shunts and alveolar death space [4, 5]. Furthermore, it has been reported that hypoxemia is associated with an increased risk of complications, such as cognitive impairment, atrial fibrillation, renal failure, and pulmonary hypertension [6, 7]. Therefore, the prevention and treatment of hypoxemia during OLV is an essential part in intraoperative anesthesia management.

A high fraction of inspiration O_2_ (FiO_2_) has been usually adopted to prevent hypoxemia in clinical practice [8]. However, a recent large multicenter cohort study found that high FiO_2_ (FiO_2_ > 60%) could promote expiratory pulmonary collapse and atelectasis, with long-term high FiO_2_ exposure being associated with worsening oxygenation index [9]. According to current protective ventilation strategies, the FiO_2_ should be controlled to the lowest level in the case of sufficient oxygenation, without specific reference of FiO_2_ concentrations [10]. Prostaglandin E1 (PEG1), a kind of selective pulmonary arterial vasodilator, has been reported to decrease pulmonary shunts and increase PaO_2_ during OLV [11], with a debated optimal dosage [12–14] Our previous study has demonstrated that PGE1 can maintain adequate oxygenation in patients under 60% FiO_2_ OLV [15]. To the best of our knowledge, the effects of PGE1 on pulmonary shunt and oxygen supply of patients under 40% FiO_2_ OLV are still unclear.

Herein, a randomized controlled trial was designed to explore the effect of PGE1 nebulization of ventilated lungs under 40% FiO_2_ OLV on maintaining adequate oxygenation based on 90 patients from the Affiliated Cancer Hospital of Nanjing Medical University between 2015 and 2017.

## Methods

### Study population

**Fig. 1:**
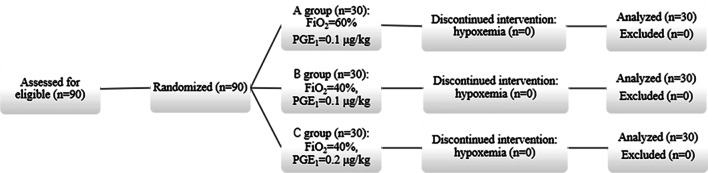
Flow diagram of study participants

Patients with EC scheduled to undergo selective open radical resection were recruited from Affiliated Cancer Hospital of Nanjing Medical University between 2015 and 2017. Diagnosis of EC patients was based on clinical, laboratory, histopathological, and gastroscopy assessment. Exclusion Criteria: (1) percutaneous oxygen saturation (SpO_2_) was below 90% during the trial and SpO_2_ did not rise to more than 90% or further reduced to less than 88% within 3 min; (2) severe arrhythmia and hemodynamic instability occurred during the whole surgery; (3) surgical duration was more than 6 h or less than 1 h; (4) liver dysfunction and kidney dysfunction were determined based on laboratory test parameters, history of coronary heart disease, cerebral infarction and glaucoma. Random number table was used for group assignment (Fig. 1): Group A (FiO_2_ = 60%, PGE1 dose = 0.1 µg/kg, n = 30), Group B (FiO_2_ = 40%, PGE1 dose = 0.1 µg/kg, n = 30), Group C (FiO_2_ = 40%, PGE1 dose = 0.2 µg/kg, n = 30). This randomized controlled clinical trial was registered at chictr.org.cn (identifier: ChiCTR1800018288, 09/09/2018), approved by the Ethics Committee of the Nanjing Medical University [2017–550].

### Anesthesia and intervention

After completing pre-operation preparation, all participants underwent general anesthesia and intubation with a left-side double-lumen tube (DLT). Thirty minutes before admission to the operating room, patients received phenobarbital 0.1 g and atropine 0.5 mg. A central venous catheter was inserted via the right internal jugular vein. Midazolam (0.05 mg/kg), fentanyl (3–4 µg/kg), propofol (1 mg/kg), and cis-atracurium (0.2 mg/kg) were adopted for anesthesia induction. A left-side DLT was then inserted, with fiber bronchoscopy being used to confirm its position. Parameters of the ventilator were set as follows: tidal volume (VT), 6–8 ml/predicted body weight; respiratory rate (RR), 12–14/min; the ratio of inspiratory to expiratory time (I:E rate), 1:2; end-tidal carbon dioxide (ETCO_2_), 35–45 mmHg; FiO_2_, 60%; positive end-expiratory pressure (PEEP), 5 cmH_2_O. The settings of VT and PEEP were kept initially unchanged throughout OLV. When the oxygen partial pressure dropped rapidly below 90%, I:E rate was adjusted to 1:1.5 by increasing RR. If SpO_2_ did not rise above 90% or further dropped below 88% within 3 min, 100% O_2_ ventilation, continuous positive airway pressure (CPAP) on non-ventilated side lungs, and resumed ventilation of double lungs were performed in sequence if necessary. The unventilated lungs were directly connected to the room air. Group B and Group C were treated with 40% FiO_2_ throughout the surgery. Depth of anesthesia was monitored by the bispectral index (BIS), keeping it between 40 and 60.

After confirming the placement of a DLT with a fiberoptic bronchoscope, the patient was repositioned to a right lateral decubitus position, and double lungs were ventilated to nebulization. All the three groups were treated with PGE1 (Beijing Tide Pharmaceutical Co., Ltd., 10 µg/2 ml, diluted to 10 ml with normal saline) nebulization to the right lung through a reconstructed breathing circuit, all nebulization being maintained for 10 min. The Yuyue 402A ultrasonic nebulizer was modified in 2 steps: firstly, seal the bottom of the sprayer tank to remove the air inlet; secondly, isolate the two original atomizer outlets to ensure that one of the exits was a new intake, and the other was the only outlet towards the patients. The device was then connected to the designated breathing circuit [16].

### Observed indicators

PaO_2_, arterial oxygen saturation (SaO_2_), SpO_2_, and arterial partial pressure of carbon dioxide (PaCO_2_) were determined from the artery and venous blood samples, which were taken at post-anesthesia/pre-nebulization (T1), OLV 10 min (T2), OLV 15 min (T3), OLV 30 min (T4), OLV 60 min (T5), OLV 120 min (T6). Mean arterial pressure (MAP) and airway pressure (PAW) were also recorded. Shunt fraction was calculated using the following formula:

$$\begin{aligned} {\text{Qs}}/{\text{Qt}} & = \left( {{\text{CcO}}_{{2}} - {\text{CaO}}_{{2}} } \right)/\left( {{\text{CcO}}_{{2}} - {\text{CvO}}_{{2}} } \right) [17, 18] \\ {\text{CaO}}_{{2}} & = {\text{PaO}}_{{2}} * 0.00{31} + \left( {{\text{Hb }}*{ 1}.{36 }*{\text{SaO}}_{{2}} } \right) \\ {\text{CvO}}_{{2}} & = \left( {{\text{PvO}}_{{2}} * 0.00{31}} \right) + \left( {{\text{Hb }}*{ 1}.{36 }*{\text{ SvO}}_{{2}} } \right) \\ {\text{CcO}}_{{2}} & = \left[ {{\text{FiO}}_{{2}} *\left( {{\text{P}}_{{\text{B}}} - {\text{P}}_{{{\text{H}}_{{2}} {\text{O}}}} } \right) - {\text{PaCO}}_{{2}} /{\text{R}}} \right]\\ &\quad*0.0031 + \left( {{\text{Hb }}*{ 1}.{36}} \right) \\ \end{aligned}$$

CcO_2_, pulmonary capillary blood oxygen content; CaO_2_, arterial blood oxygen content; CvO_2_, mixed venous blood oxygen content; PvO_2_, mixed venous oxygen partial pressure; SvO_2_, mixed venous oxygen saturation, we replaced SvO_2_ with central venous oxygen saturation (ScvO_2_); P_B_, atmospheric pressure (760 mmHg); P_H2O_, 37 °C water vapor pressure (47 mmHg); R, respiratory quotient (0.8).

### Measurement of serum malondialdehyde (MDA) and superoxide dismutase (SOD), Tumor necrosis factor-α (TNF-α), and interleukin 6 (IL-6)

Venous blood sampling through the central line was collected at T1, T5, 30 min after restarting two-lung ventilation (TLV) (T7) and 24 h after surgery (T8). After centrifugation (3000 rpm, 4 °C, 20 min), the serum was stored at − 80 °C until being assayed. According to the manufacturer’s instructions, the concentrations of MDA, SOD, TNF-α, and IL-6 were measured by human MDA, SOD, TNF-α, and IL-6 ELISA Kits.

### Postoperative characteristics

Clinical pulmonary infection score (CPIS) [19], complications (pleural effusion, pneumothorax, anastomotic leakage and incision infection), length of stay in the hospital or ICU, and the grade of wound healing were used as our prognostic indicators.

### Outcomes

The primary outcomes were oxygenation and pulmonary shunt during OLV, including the observed and measurement indicators of PaO_2_, PaO_2_/FiO_2_ and Qs/Qt, which were determined at T2-T6. Secondary outcomes were oxidative stress after OLV, including MDA, SOD, TNF-α, and IL-6 at T1, T5, T7 and T8.

### Statistical analysis

All data analyses were performed by R4.0.3. At least 24 patients were required in each group to achieve a power of 0.8 and a two-sided Alpha level of 0.05. Thirty patients were included in each group considering the drop-out rate of 20%. The measurement data were expressed as mean ± standard deviation (M ± SD) or median [interquartile range, M (Q_1_, Q_3_)], and intergroup analysis was made by one-way ANOVA, Kruskal–Wallis rank test, or repeated-measures ANOVA of three factors, as appropriate. In addition, Student–Newman–Keuls (SNK) was used for comparisons between groups at each time point. Counting data was tested by the chi-square test. *P* < 0.05 was considered statistically significant.

## Results

### Baseline characteristics

A total of 90 patients [71 (78.89%) male, 19 (21.11%) females] were enrolled and randomly divided into three groups, with an average age of (63.32 ± 6.46) years old (Table 1). No patient developed hypoxemia among the two groups. No significant differences in gender, age, BMI, duration of surgery, and duration of OLV were observed among those patients (*P* > 0.05). Table 2 shows the clinical characteristics of all the patients.

**Table 1:** Basic situation of studied patients [n (%)/Mean ± SD]

Characteristic	All (n = 90)
Gender, n (%)	
Male	71 (78.89)
Female	19 (21.11)
Age, year	63.32 ± 6.46
BMI, kg/m^2^	23.16 ± 2.91
Duration of surgery, min	209.20 ± 60.92
Duration of OLV, min	173.57 ± 50.75

BMI, Body mass index; OLV, one-lung ventilation

**Table 2:** Characteristic of patients with esophageal cancer [n (%)/Mean ± SD]

Characteristic	All (n = 90)	A group (n = 30)	C group (n = 30)	B group (n = 30)	*P*
Gender					0.627
Male	71 (78.89)	25 (83.33)	22 (73.33)	24 (80.00)	
Female	19 (21.11)	5 (16.67)	8 (26.67)	6 (20.00)	
Age, year	63.32 ± 6.46	63.20 ± 6.03	64.60 ± 5.91	62.17 ± 7.33	0.346
BMI, kg/m^2^	23.16 ± 2.91	22.60 ± 3.21	23.80 ± 2.55	23.08 ± 2.88	0.276
Duration of surgery, min	209.20 ± 60.92	213.53 ± 66.25	192.23 ± 36.02	221.83 ± 72.26	0.152
Duration of OLV, min	173.57 ± 50.75	177.40 ± 43.84	159.90 ± 39.30	183.40 ± 64.26	0.177

BMI, Body mass index; OLV, one-lung ventilation

A, 60% FiO_2_ + 0.1 µg/kg PGE_1_; B, 40% FiO_2_ + 0.1 µg/kg PGE_1_; C, 40% FiO_2_ + 0.2 µg/kg PGE_1_

### Effects of PGE1 nebulization of ventilated lungs under different FiO_2_ OLV on oxygenation and pulmonary shunt

Data acquired at each time point were statistically analyzed using analysis of variance. The results showed that, during OLV, patients in Group C and B had lower levels of PaO_2_, SaO_2_, SpO_2_, MAP, and Qs/Qt than those in Group A (Table 3). At T2 (OLV 10 min), patients in Group C and B exhibited a lower PaO_2_/FiO_2_ than those in Group A, without any statistical difference at other time points (Table 3).

**Table 3:** The levels of PaO_2_, PaO_2_/FiO_2_, SaO_2_, SPO_2_, ETCO_2_, PaCO_2_, MAP, PAW and QS/QT among three groups

Indicator	Group	T1	T2	T3	T4	T5	T6
PaO_2*_, mmHg							
	A	271.5 ± 37.5	153.4 ± 32.2	132.0 ± 28.5	121.0 ± 27.3	115.4 ± 34.9	153.4 ± 45.3
	B	175.0 ± 51.1^a^	88.7 ± 27.6^a^	89.2 ± 29.4^a^	74.4 ± 17.8^a^	81.4 ± 21.4^a^	99.5 ± 25.0^a^
	C	172.5 ± 90.8^a^	84.9 ± 17.1^a^	82.3 ± 15.9^a^	76.5 ± 17.5^a^	80.1 ± 25.3^a^	101.1 ± 22.0^a^
PaO_2_/FiO_2_, mmHg							
	A	452.5 ± 62.4	255.6 ± 53.6	219.9 ± 47.5	201.8 ± 45.5	192.4 ± 58.3	255.6 ± 75.6
	B	437.4 ± 127.8	221.8 ± 69.0^a^	222.9 ± 73.6	185.9 ± 44.4	203.5 ± 53.5	248.9 ± 62.5
	C	397.6 ± 142.8	212.3 ± 42.8^a^	205.7 ± 39.7	191.3 ± 43.9	200.2 ± 63.4	252.8 ± 54.9
SaO_2_, %							
	A	100.0 ± 0.0	98.8 ± 0.9	98.4 ± 1.3	97.5 ± 1.5	97.9 ± 1.4	98.9 ± 1.4
	B	99.3 ± 0.7	95.2 ± 2.4^a^	95.2 ± 2.9^a^	93.6 ± 3.1^a^	95.0 ± 2.1^a^	97.2 ± 1.7^a^
	C	98.6 ± 2.3	95.4 ± 2.8^a^	94.8 ± 2.6^a^	93.9 ± 3.0^a^	94.5 ± 2.8^a^	97.2 ± 1.7^a^
SpO_2_, %							
	A	99.5 ± 0.7	99.1 ± 1.1	98.2 ± 3.0	98.3 ± 1.5	98.3 ± 1.5	99.4 ± 0.9
	B	99.2 ± 1.2	97.1 ± 2.2^a^	96.6 ± 2.1^a^	95.2 ± 2.4^a^	96.3 ± 2.1^a^	98.1 ± 1.5
	C	98.0 ± 5.9	96.4 ± 4.1^a^	95.4 ± 4.1^a^	94.6 ± 4.3^a^	95.3 ± 3.5^a^	97.4 ± 4.1
PaCO_2_, mmHg							
	A	42.9 ± 6.0	43.3 ± 6.2	41.8 ± 5.8	40.1 ± 5.8	41.6 ± 6.3	39.5 ± 6.8
	B	46.2 ± 18.1	46.3 ± 7.2	43.8 ± 4.9	42.5 ± 5.2	40.9 ± 6.1	39.4 ± 4.7
	C	43.4 ± 4.9	48.9 ± 8.4	47.1 ± 7.8	42.6 ± 6.0	40.3 ± 5.6	39.6 ± 6.0
ETCO_2_, mmHg							
	A	35.4 ± 5.8	36.8 ± 4.6	35.7 ± 4.1	34.7 ± 4.0	35.1 ± 4.4	34.4 ± 4.4
	B	35.2 ± 3.6	36.3 ± 4.7	35.6 ± 4.0	33.9 ± 4.6	33.1 ± 4.4	33.6 ± 3.3
	C	35.8 ± 5.9	38.3 ± 7.4	37.7 ± 7.5	35.1 ± 6.0	33.7 ± 6.3	33.8 ± 6.5
MAP_*_, mmHg							
	A	102.1 ± 14.7	96.5 ± 16.0	92.6 ± 16.3	94.1 ± 22.9	95.6 ± 21.3	89.3 ± 26.5
	B	72.6 ± 9.1^a^	76.8 ± 11.5^a^	78.4 ± 13.5^a^	77.2 ± 11.8^a^	70.3 ± 11.2^a^	67.5 ± 10.1^a^
	C	77.3 ± 12.1^a^	83.9 ± 11.8^ab^	84.8 ± 12.0^ab^	80.8 ± 11.1^a^	72.3 ± 9.5^a^	68.7 ± 10.5^a^
PAW, cmH_2_O							
	A	14.9 ± 3.2	22.5 ± 3.7	23.0 ± 3.4	23.6 ± 4.5	24.3 ± 4.5	24.3 ± 3.9
	B	15.1 ± 3.7	21.9 ± 4.9	21.4 ± 4.9	22.1 ± 5.9	21.8 ± 4.9	22.0 ± 3.5
	C	16.6 ± 4.6	22.1 ± 4.2	22.1 ± 3.9	22.7 ± 4.0	23.2 ± 3.8	24.1 ± 5.0
Qs/Qt_*_							
	A	11.5 ± 1.7	16.8 ± 1.4	17.8 ± 1.2	18.4 ± 1.2	18.5 ± 1.5	17.0 ± 1.9
	B	7.8 ± 2.4^a^	14.4 ± 2.6^a^	14.7 ± 2.0^a^	16.0 ± 2.0^a^	15.7 ± 2.1^a^	15.8 ± 1.4^a^
	C	7.8 ± 2.6^a^	14.0 ± 2.9^a^	14.5 ± 2.4^a^	15.2 ± 1.6^a^	16.2 ± 1.6^a^	15.3 ± 2.2^a^

^a^*P* < 0.05 compared with Group A; ^b^*P* < 0.05 compared with Group B; *there is a difference at T1. (baseline), and the subsequent time points are adjusted with the level at T1

T1, pre-nebulization, T2, one-lung ventilation (OLV) 10 min, T3: OLV 15 min, T4: OLV 30 min, T5: OLV 60 min, T6: OLV 120 min

PaO_2_, partial pressure of arterial oxygen; FiO_2_, fraction of inspiration O_2_; SaO_2_, arterial oxygen saturation; SpO_2_, percutaneous oxygen saturation; PaCO_2_, arterial partial pressure of carbon dioxide; ETCO_2_, end-tidal carbon dioxide; MAP, mean arterial pressure; PAW, airway pressure; A, 60% FiO_2_ + 0.1 µg/kg PGE_1_; B, 40% FiO_2_ + 0.1 µg/kg PGE_1_; C, 40% FiO_2_ + 0.2 µg/kg PGE_1_

### Effects of PGE1 nebulization of ventilated lungs under different FiO_2_ OLV on inflammatory factors and postoperative characteristics

**Fig. 2:**
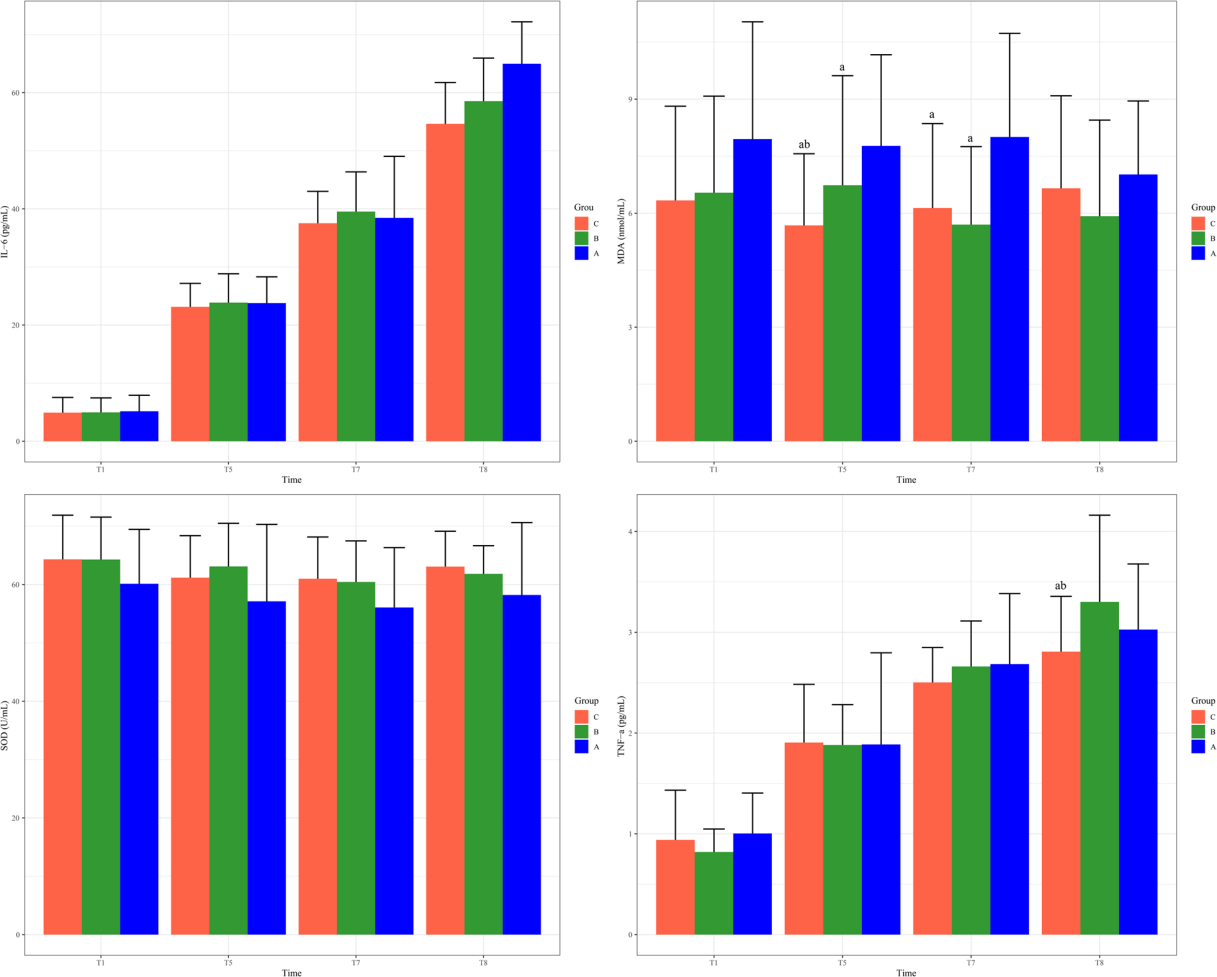
IL-6 (**A**), MDA (**B**), TNF-α (**C**), and SOD (**D**) levels of patients in different time points and different groups. **A** 60% FiO_2_ + 0.1 µg/kg PGE_1_; **B** 40% FiO_2_ + 0.1 µg/kg PGE_1_; **C** 40% FiO_2_ + 0.2 µg/kg PGE_1_.T1, pre-nebulization; T5, one-lung ventilation (OLV) 60 min, T7, two-lung ventilation (TLV) 30 min; T8, 24 h after surgery. ^a^*P* < 0.05 compared with Group A; ^b^*P* < 0.05 compared with Group B

Data of inflammatory factors collected at each time point were compared by analysis of variance. The results presented that the IL-6 levels of patients in different groups were different at T8 (F = 3.431, *P* = 0.038), with the level of IL-6 in Group C being lower than that of Group B and A (Table 4 and Fig. 2A). MDA levels among the three groups were different at T5 (F = 4.692, *P* = 0.012) and T7 (F = 5.906, *P* = 0.004), with the MDA level of Group C being lower than that of Group B and A at T5, and the MDA level of Group C and B being lower than that of Group A at T7 (Table 4 and Fig. 2B). In terms of TNF-α level, patients in Group C had a lower level than Group B and A at T8 (F = 3.598, *P* = 0.033) (Table 4 and Fig. 2C). Differences in the level of SOD were not statistically significant (Table 4 and Fig. 2D). Regarding postoperative characteristics, the lung infection scores of patients in Group C and B were lower than those of Group A, and the difference was statistically significant (*P* < 0.05) (Table 5). In addition, there was no significant difference in lung infection scores, complications, length of stay in hospital and ICU, and grade of wound healing between patients in Group C and those who did not use PGE1 (Table 6).

**Table 4:** Levels of inflammatory biomarkers

Biomarkers	Group	T1	T5	T7	T8
IL-6, pg/mL	A	5.15 ± 2.76	23.78 ± 4.51	38.45 ± 10.59	64.97 ± 7.22
	B	4.96 ± 2.49	23.86 ± 4.97	39.53 ± 6.84	58.54 ± 7.42^a^
	C	4.92 ± 2.62	23.13 ± 4.04	37.52 ± 5.49	54.63 ± 7.11^ab^
MDA, nmol/mL	A	7.95 ± 3.08	7.77 ± 2.40	8.00 ± 2.73	7.02 ± 1.93
	B	6.54 ± 2.54	6.73 ± 2.88^a^	5.7 ± 2.05^a^	5.92 ± 2.53
	C	6.34 ± 2.48	5.68 ± 1.88^ab^	5.68 ± 2.22^a^	6.65 ± 2.43
SOD, U/mL	A	60.13 ± 9.32	57.12 ± 13.18	56.06 ± 10.26	58.21 ± 12.41
	B	64.29 ± 7.28	63.09 ± 7.4	60.44 ± 7.05	61.83 ± 4.83
	C	64.32 ± 7.57	61.17 ± 7.2	60.99 ± 7.16	63.07 ± 6.07
TNF-α, pg/mL	A	1.00 ± 0.40	1.89 ± 0.91	2.68 ± 0.70	3.03 ± 0.65
	B	0.82 ± 0.23	1.88 ± 0.4	2.66 ± 0.45	3.3 ± 0.86
	C	0.94 ± 0.49	1.91 ± 0.58	2.5 ± 0.35	2.81 ± 0.55^ab^

^a^*P* < 0.05 compared with Group A; ^b^*P* < 0.05 compared with Group B

IL-6, interleukin-6; MDA, malondialdehyde; SOD, superoxide dismutase; TNF-α, tumor necrosis factor-α

T1, pre-nebulization; T5, OLV 60 min; T7, TLV 30 min; T8, 24 h after surgery

A, 60% FiO_2_ + 0.1 µg/kg PGE_1_; B, 40% FiO_2_ + 0.1 µg/kg PGE_1_; C, 40% FiO_2_ + 0.2 µg/kg PGE_1_

**Table 5:** Postoperative characteristics of patients in Group A, C and B [M (Q_1_, Q_3_)/n (%)]

Characteristics	All (n = 90)	A group (n = 30)	B group (n = 30)	C group (n = 30)	*P*
Score for lung infection	2.53 (1.6, 4.5)	4.0 (3.0, 4.7)	1.0 (1.0, 2.7)^*^	2.0 (1.0, 3.0)^*^	< 0.001
Complication					
Pleural effusion	2 (2.22)	0 (0.00)	2 (6.67)	0 (0.00)	0.326
Pneumothorax	1 (1.11)	0 (0.00)	0 (0.00)	1 (3.33)	1.000
Anastomotic leakage	3 (3.33)	0 (0.00)	2 (6.67)	1 (3.33)	0.770
Incision infection	1 (1.11)	0 (0.00)	0 (0.00)	1 (3.33)	1.000
LOS in hospital, day	21.5 (19.0, 25.7)	21.0 (18.3, 21.0)	21.0 (19.0, 24.7)	22.0 (19.0, 25.7)	0.175
LOS in ICU, day	1.0 (1.0, 1.0)	1.0 (1.0, 1.0)	1.0 (1.0, 1.0)	1.0 (1.0, 1.0)	0.372
Grade of wound healing					0.318
1	86 (95.56)	30 (100.00)	29 (96.67)	27 (90.00)	
≥ 2	4 (4.44)	0 (0.00)	1 (3.33)	3 (10.00)	

^*^P < 0.05 compared with Group A

LOS, Length of stay

A, 60% FiO_2_ + 0.1 µg/kg PGE_1_; B, 40% FiO_2_ + 0.1 µg/kg PGE_1_; C, 40% FiO_2_ + 0.2 µg/kg PGE_1_

**Table 6:** Postoperative characteristics of patients in Group C and those who did not use PGE1 under 40% FiO_2_ [M (Q_1_, Q_3_)/n (%)]

Characteristics	40% FiO_2_ + 0 PGE1 (n = 30)	C group (n = 30)	*P*
Score for lung infection	2.00 (1.00,3.00)	2.00 (1.00, 3.00)	0.331
Complication			
Pneumothorax	0 (0.00)	1 (3.33)	1.000
Anastomotic leakage	3 (10.00)	1 (3.33)	0.612
Incision infection	0 (0.00)	1 (3.33)	1.000
LOS in hospital, day	21.00 (19.00,28.00)	22.00 (19.00, 26.00)	0.894
LOS in ICU, day	1.00 ± 0.00	1.03 ± 0.18	0.326
Grade of wound healing			0.321
1	28 (96.55)	27 (90.00)	
2	1 (3.45)	2 (6.67)	
3	0 (0.00)	1 (3.33)	

LOS, Length of stay

C, 40% FiO_2_ + 0.2 µg/kg PGE_1_

## Discussion

PGE1 has been reported to maintain adequate oxygenation among patients under 60% FiO_2_ one-lung ventilation (OLV) [15]. However, the effects of PGE1 on patients under 40% FiO_2_ OLV remain uncertain. We intended to study the effects of PGE1 on pulmonary shunt and oxygenation of patients under 40% FiO_2_ OLV and provide a reference concentration of PGE1. Although the levels of PaO_2_, SaO_2_, MAP, and SaO_2_ decreased when the concentration of FiO_2_ decreased from 60 to 40%, PaO_2_ and SaO_2_ remained at a safe level. Furthermore, no statistical difference among patients treated with 60% FiO_2_ and 40% FiO_2_ was observed regarding PaCO_2_, ETCO_2_, and PAW levels. In addition, the level of PaO_2_/FiO_2_ decreased at T2, but the difference at other moments was not statistically significant, with Qs/Qt of patients treated with 40% FiO_2_ being lower than 60%. Those results indicated that the concentration of FiO_2_ could be reduced from 60 to 40%. In addition, among patients treated with 40% FiO_2_, increasing the concentration of PGE1 could reduce the levels of IL-6, MDA, and TNF-α. 40%FiO_2_ + 0.2 µg/kg PGE1 is recommended as a better combination on account of its effects on the inflammatory factors.

Previous research illustrated that 58% of patients under 50% FiO_2_ OLV had hypoxemia and needed higher FiO_2_ to maintain 95% SpO_2_ [20]. However, our result showed that no patient (FiO_2_ = 40% + 0.2 µg/kg PGE1) developed hypoxemia, indicating that PGE1 could be conducive to maintaining adequate oxygenation under 40% FiO_2_ OLV.

During OLV, blood flow is distributed in the collapsed lung without ventilation, and the ventilation/perfusion ratio (V/Q) is almost zero, which results in pulmonary blood flow, without oxygenation, flowing directly into the left atrium and then into the systemic circulation, causing pulmonary shunt [14]. This study showed that once OLV was initiated, the patients’ level of Qs/Qt declined compared to before, which is consistent with the previous research. Inhaling PEG1, a kind of selective pulmonary artery dilator, by ultrasonic atomization during OLV could dilate the pulmonary artery, which causes stealing blood from the shunt area to the non-shunt area, reducing intrapulmonary shunt [21, 22]. In addition, a previous study demonstrated that PGE1 could decrease pulmonary shunt and increase PaO_2_ in a dose-dependent manner during OLV [11]. The results showed that the Qs/Qt of patients using 0.2 µg/kg PGE1 and 0.1 µg/kg PGE1 under the condition of 40% FiO_2_ was significantly lower than that of patients using 0.1 µg/kg PGE1 under 60% FiO_2_. This may be because pre-nebulization of PGE1 to the ventilated lung of OLV during two-lung ventilation could reduce shunt and improve oxygenation. However, our results showed that there was no statistical difference in the levels of Qs/Qt and PaO_2_/FiO_2_ between patients treated by 0.2 µg/kg PGE1 and 0.1 µg/kg PGE1 under 40% FiO_2_, suggesting that 0.1 µg/kg and 0.2 µg/kg PGE1 would maintain sufficient oxygenation, and further studies are needed to explore whether the mechanism of PGE1 is different under different concentrations of FiO_2_. In a word, our research results showed that even under 40% FiO_2_ OLV, PGE1 could keep lower pulmonary shunt. The mechanisms may be multifaceted. It has been proved experimentally that hypoxic pulmonary vasoconstriction (HPV) could improve oxygenation function in vivo by reducing intrapulmonary shunt [23, 24]. We speculate that PGE1 might affect Qs/Qt by regulating HPV.

Patients treated with 40% FiO_2_ had lower levels of IL-6 and MDA than those who used 60% FiO_2_. In addition, we found that patients receiving 0.2 µg/kg PGE1 had lower levels of IL-6, MDA, and TNF-α than those receiving 0.1 µg/kg PGE1 under 40% FiO_2_. Previous research exhibited that MDA levels were indirect indicators of oxidative stress, and MDA was associated with lung injury [25]. Animal experiments and clinical studies showed that intravenous infusion of PGE1 could lower serum IL-6 and TNF-α levels, reducing organ inflammatory damage [26, 27]. Considering the effects of the inflammatory factors mentioned, 40% FiO_2_ + 0.2 µg/kg PGE1 is recommended as a better combination, which may decrease the risk of infection at the time point we have observed.

Prior research indicated that 60% FiO_2_ + 0.1 µg/kg PGE1 could maintain adequate oxygenation during OLV, improving oxidative stress and complications after OLV. This study further found that 0.1 µg/kg and 0.2 µg/kg PGE1 could be adopted to maintain the patients’ oxygenation under 40% FiO_2_. Additionally, patients under 40% FiO_2_ could benefit more from 0.2 µg/kg PGE1 than from 0.1 µg/kg PGE1 in terms of inflammatory factors.

## Limitation

Nevertheless, several limitations in this study could not be ignored. Firstly, further subgroup analysis based on complications, age, or gender was unable to be performed due to the small sample size. Secondly, participants with pulmonary dysfunction were not be enrolled in this study. Whether the recommended combination (40% FiO_2_ + 0.2 µg/kg PGE1) is suitable for different populations remains to be further verified. Thirdly, we used ScvO_2_ instead of SvO_2_ in the calculation of shunt fraction. ScvO_2_ measures the oxygen saturation of the superior vena cava, while SvO_2_ measures the oxygen saturation of the whole body, including the abdomen and lower extremities, so the absolute value is different. Using ScvO_2_ to estimate SvO_2_ in calculating the shunt fraction was imperfect, and ScvO_2_ depended on catheter placement, patient anatomy and physiological state [17]. Fourthly, we speculated that pre-nebulization of PGE1 to the ventilated lung of OLV during two-lung ventilation could reduce shunt and improve oxygenation, but FiO_2_ changes may also have an impact on the shunt. Based on the existing results in this article, we are not sure whether the reduction in the shunt fraction is affected by PGE1. More rigorously designed prospective researches with larger sample sizes are required to confirm our results.

## Conclusion

PGE1 nebulized on the ventilated lung and FiO_2_ reduced to 40% for OLV could reduce the shunt fraction and maintain oxygenation. We recommend a PGE1 concentration of 0.2 µg/kg, which could reduce the levels of inflammatory factors IL-6, MDA and TNF-α during surgery to a certain extent.

## Data Availability

The datasets used and/or analyzed during the current study are available from the corresponding author on reasonable request.

## Abbreviations

PGE1: Prostaglandin E1
OLV: One-lung ventilation
FiO_2_: Fraction of inspiration O_2_
EC: Esophageal cancer
PaO_2_: Partial pressure of arterial oxygen
DLT: Double-lumen tube
VT: Tidal volume
RR: Respiratory rate
ETCO_2_: End-tidal carbon dioxide
PEEP: Positive end-expiratory pressure
BIS: Bispectral index
SaO_2_: Arterial oxygen saturation
SpO_2_: Percutaneous oxygen saturation
PaCO_2_: Arterial partial pressure of carbon dioxide
MAP: Mean arterial pressure
PAW: Airway pressure
CcO_2_: Pulmonary capillary blood oxygen content
CaO_2_: Arterial blood oxygen content
CvO_2_: Mixed venous blood oxygen content
PvO_2_: Mixed venous oxygen partial pressure
SvO_2_: Mixed venous oxygen saturation
ScvO_2_: Central venous oxygen saturation
P_B_: Atmospheric pressure
P_H2O_: 37 °C water vapor pressure
R: Respiratory quotient
MDA: Malondialdehyde
SOD: Superoxide dismutase
TNF-α: Tumor necrosis factor-α
IL-6: Interleukin 6
TLV: Two-lung ventilation
CPIS: Clinical pulmonary infection score
M ± SD: Mean ± standard deviation
SNK: Student–Newman–Keuls
